# Nucleus-specific X-ray stain for 3D virtual histology

**DOI:** 10.1038/s41598-018-36067-y

**Published:** 2018-12-14

**Authors:** Mark Müller, Melanie A. Kimm, Simone Ferstl, Sebastian Allner, Klaus Achterhold, Julia Herzen, Franz Pfeiffer, Madleen Busse

**Affiliations:** 1 Department of Physics and Munich School of BioEngineering, Technical University of Munich, 85748 Garching, Germany; 2 Department of Diagnostic and Interventional Radiology, Klinikum rechts der Isar, Technical University of Munich, 81675 Munich, Germany

## Abstract

Histological investigations are indispensable with regards to the identification of structural tissue details but are limited to two-dimensional images, which are often visualized in one and the same plane for comparison reasons. Nondestructive three-dimensional technologies such as X-ray micro- and nanoCT have proven to provide valuable benefits for the understanding of anatomical structures as they allow visualization of structural details in 3D and from arbitrary viewing angles. Nevertheless, low attenuation of soft tissue has hampered their application in the field of 3D virtual histology. We present a hematein-based X-ray staining method that specifically targets the cell nuclei of cells, as demonstrated for a whole liver lobule of a mouse. Combining the novel staining protocol with the high resolving power of a recently developed nanoCT system enables the 3D visualization of tissue architecture in the nanometer range, thereby revealing the real 3D morphology and spatial distribution of the cell nuclei. Furthermore, our technique is compatible with conventional histology, as microscopic slides can be derived from the very same stained soft-tissue sample and further counter staining is possible. Thus, our methodology demonstrates future applicability for modern histopathology using laboratory X-ray CT devices.

## Introduction

Understanding how tissue architecture is organized from the cellular level to the tissue scale, is a key to next generation medicinal diagnosis^1^. The current gold standard to an accurate diagnosis is histology, which is a technique limited to two dimensions. Very thin microscopic slides (2–10 µm thick cuts) obtained from a 3D biopsy are investigated using conventional and modern immunohistochemical and histological staining techniques^2^.

To allow for a more detailed systems biology analysis of tissue, an accurate 3D reconstruction is mandatory. Micro- and nanoCT are powerful tools holding the potential to provide an accurate 3D reconstruction of tissue. Developments on the technological side allow for comparative resolution to 2D conventional histology with devices ranging from large particle accelerators^3–5^ to laboratory X-ray devices^6–10^. Next to the technological requirements needed for attenuation-based X-ray microscopy, X-ray suitable staining agents such as phosphotungstic acid (PTA), iodine potassium iodide (IKI) or iodine in ethanol (I2E)/iodine in methanol (I2M) are an important aspect^11–16^. Nonetheless, the availability of staining agents that (i) target a specific biological morphology, (ii) stain homogenously and completely, (iii) are easy to handle, (iv) are speedily penetrating the tissue without creating artefacts such as diffusion rings, (v) are suitable for large and dense tissue samples, and (vi) are fully compatible with histology, is currently very limited^17^. Next to the cell cytoplasm, the cell nuclei are of significance for a first diagnosis concerning mostly the general morphological appearance. Almost every histological sample is stained with the standard hematoxylin and eosin (H&E) protocol. Although, it should be mentioned that ‘standard’ is not obvious for the hematoxylin stain as many different protocols exist, which result from different tissue or pre-treatment parameters^18^. Recently, we have presented an X-ray tailored eosin-based staining method, which interacts with the cell cytoplasm^17^. Within this work, we introduce a hematein-based staining method, which has been explicitly developed for CT allowing now for a direct 3D visualization of cell nuclei within soft-tissue samples. The powerful potential of microCT and nanoCT to enable future insights into tissue organization, and thus, systems biological analysis, is demonstrated on a mouse liver lobule. In general, the microscopic recognition of the tissue architecture may lead to the understanding of widespread diseases such as osteoarthritis and cancer in the future^19^.

## Results

### Development of a hematein-based X-ray stain

To demonstrate the need for the development of a new X-ray suitable hematein-based staining protocol, the commonly used hematoxylin stains were applied as 0.1% [weight/volume (w/v)] for Mayer’s hematoxylin or 0.5% (w/v) for Weigert’s iron hematoxylin aqueous solution to mouse liver tissue. To test the contrast enhancement by the staining protocols, the very same mouse liver lobule was used for X-ray CT imaging before and after staining using the same imaging parameters (one mouse liver lobule for each staining procedure). A desirable contrast enhancement was not achieved (Supplementary Fig. S1 and staining protocols used).

**Figure 1:**
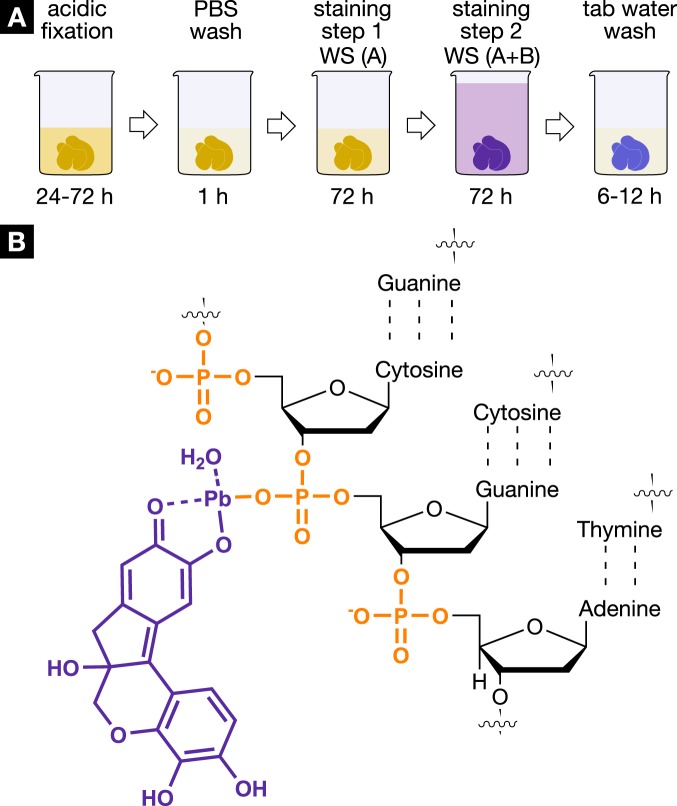
Staining protocol and interaction of the hematein-based X-ray stain with soft tissue. (**A**) The developed hematein-based staining procedure shows the individual steps involved including incubation and staining times. Staining step 1 was conducted using lead(II) acetate trihydrate as the heavy metal source. The lead(II) acetate trihydrate was dissolved in distilled water (c = 666 mM) and is referred to as working solution (**A**) (WS (A)). The staining step 2 involved a hematein solution in absolute ethanol (WS (**B**), 10% (w/v); c = 333 mM), which was derived from hematoxylin and was added to WS (A). More details concerning the entire staining protocol are described in the Materials and Methods section. (**B**) The positively charged hematein lead(II) complex (purple), which is built *in situ* in the soft-tissue sample, is interacting with the negatively charged phosphate backbone of the DNA (orange) present in the nucleus of the cell. The selective interaction of the hematein lead(II) complex with the DNA is achieved by acidification of the soft tissue during fixation or before staining and allows for a higher accumulation of the hematein lead(II) complex within the cell nucleus.

To improve the contrast enhancement within the soft tissue, the metal ion was exchanged with the much higher atomic number element lead(II) (Z = 82). Several concentrations of the hematein lead(II) complex were tested (with maximum solubility of hematein in absolute ethanol as end point). The highest hematein lead(II) complex concentration resulted in the best contrast enhancement within the soft tissue as it was expected according to the Lambert-Beer Law. Therefore, the final staining protocol was carried out with the highest concentration. Further improvements to reach the final staining protocol, which consists of five steps, were made (Fig. 1A). Here, the acidification of the soft-tissue sample during fixation or before staining is crucial. The soft tissue is optimally prepared on molecular level for the staining procedure with the hematein lead(II) complex. Functional groups present within the cell cytoplasm, e.g. amino -, hydroxy - or thiol groups, are protonated, which allows for repulsion of the positively charged hematein lead(II) complex and optimal targeting of the cell nuclei present within the soft-tissue sample. Thus, leading to a higher accumulation of staining agent within the cell nuclei by strengthening the ionic interaction of the positively charged hematein lead(II) complex with the negatively charged phosphate backbone of the deoxyribonucleic acid (DNA) (Fig. 1B) to form a hematein lead(II) DNA complex. A summary of parameters and conditions used for the development and optimization of the X-ray suitable hematein staining protocol is provided in the supplementary information (see Table S1).

### MicroCT investigations of hematein stained mouse liver tissue

**Figure 2:**
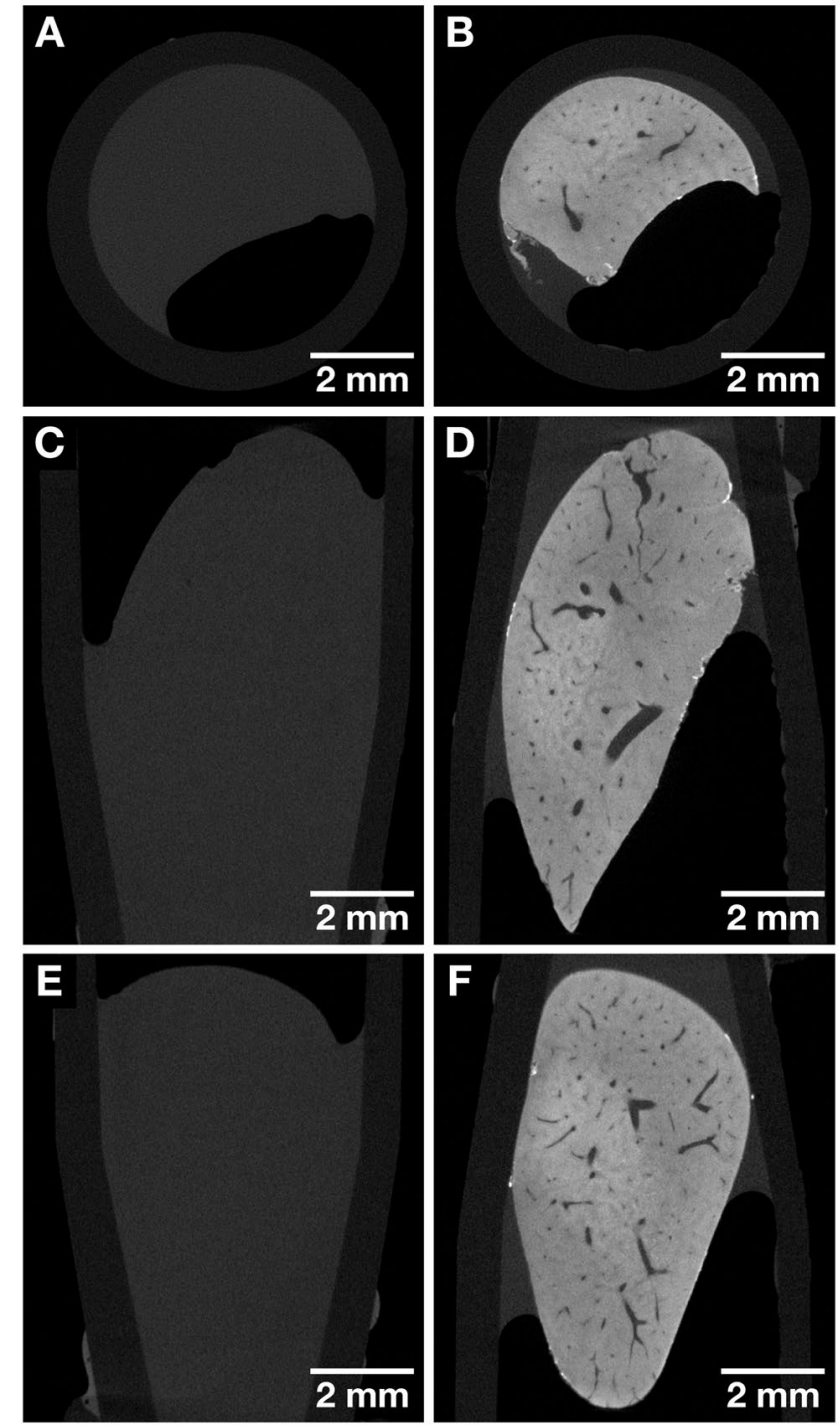
CT slices of the same whole mouse liver lobule before and after staining highlighting the contrast enhancement obtained after application of the hematein-based X-ray stain. Both data sets were acquired with the Xradia Versa 500 microCT using identical acquisition parameters. The voxel size in both data sets is 13.5 µm. (**A**, **C** and **E**) Overview images of the unstained mouse liver lobule representing the views along the Cartesian axes. (**B**, **D** and **F**) Overview images of the same mouse liver lobule sample in (**A**, **C** and **E**) after staining representing the views along the Cartesian axes. Anatomical structures such as the vasculature are visualized.

Before staining was applied using the final version of the hematein-based staining protocol, a mouse liver lobule was imaged with microCT. Anatomical structural information was not visible (Fig. 2A, C and E). The very same mouse liver lobule was subjected to the developed hematein-based staining protocol and again imaged with the microCT. The actual staining occurs in two steps, whereby the hematein lead(II) complex is built *in situ* in the soft tissue. The metal precursor, here lead(II) acetate trihydrate, is applied first, followed by staining with the hematein alcoholic solution. The tab water wash after staining is crucial for the specific cell nuclei staining result. The overview microCT scan provided the desired contrast enhancement (Fig. 2B, D and F). A clear distinction of anatomical structures such as the vasculature was achieved. Furthermore, the staining was homogeneous within the entire mouse liver lobule, which is not self-evident for large liver tissue samples^20^. Figure 2 highlights already one benefit of 3D imaging, namely the accessibility of a series of CT slices in arbitrary planes, which allows to view the soft-tissue sample from different viewing angles. This possibility is not given in conventional 2D histology, as here the viewing plane is set upon embedding of the soft-tissue sample in paraffin. To allow for comparison between different soft-tissue samples embedding of the sample occurs often the same way. Thus, to the pathologist usually only one plane is known.

### NanoCT investigations of hematein stained mouse liver tissue

**Figure 3:**
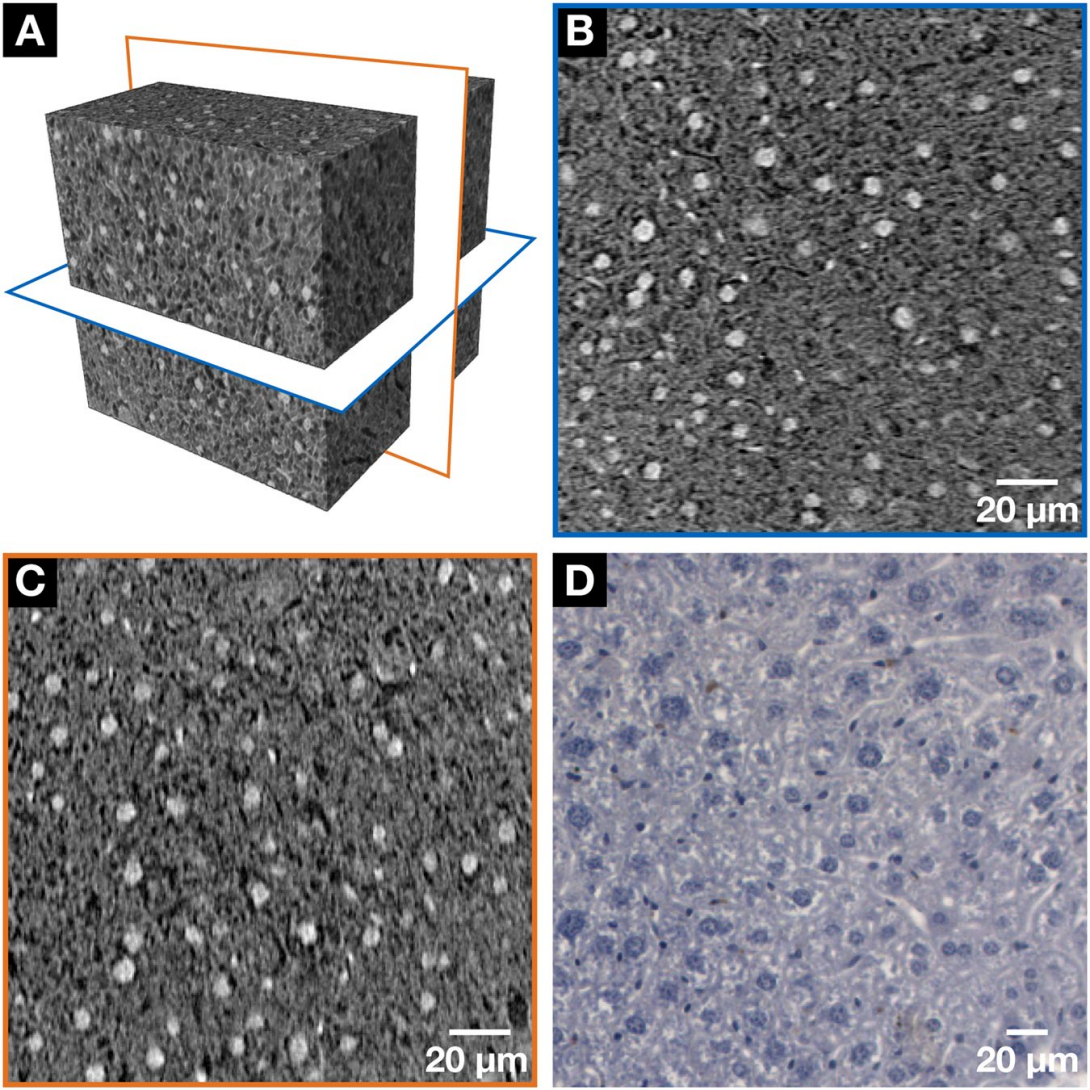
NanoCT data (**A–C**) in comparison with the histological microscopic slide (**D**) derived from the same mouse liver lobule after application of the hematein-based X-ray staining protocol. Clear visualization of the larger hepatocyte cell nuclei and the smaller cell nuclei such as Kupffer cells and SECs in white (**A–C**) or dark purple (**D**) and the BC network displayed in black (**A-C**) or white (**D**) was achieved, respectively. (**A**) The volume of interest (VOI) highlighting the two nanoCT slices shown in (**B**, blue frame) and (**C**, orange frame). (**B**, **C**) Representative individual nanoCT slices as indicated in the VOI from (**A**). (**B**) and (**C**) are positioned orthogonal to each other. The orientation of the BC network, which is formed by the hepatocytes is seen, i.e. more horizontal arrangement is seen in (**B**) and a more vertical alignment in (**C**). The nanoCT slice thickness is 580 nm. (**D**) Representative histological microscopic slide with a thickness of 3 µm obtained from the same mouse liver lobule sample after the applied hematein-based staining and embedding in a paraffin block.

To investigate the tissue on (sub)-cellular level, smaller tissue pieces from the very same mouse liver lobule were dissected and analyzed with the nanoCT. The results obtained are visualized in Fig. 3, whereby Fig. 3A shows the volume of interest (VOI) where the two nanoCT slices (thickness of 580 nm) located orthogonal to each other were chosen for a more detailed look (Fig. 3B, C). Here, the cell nuclei of the hepatocytes as well as the cell nuclei containing other typical cell types such as Kupffer cells and sinusoidal endothelial cells (SECs) are clearly highlighted. The black-appearing whole-like structures represent the bile canalicular (BC) network while the darker gray-values indicate the cytoplasm. The orientation of the BC network, which is formed by the hepatocytes is seen. The nanoCT slice seen in Fig. 3B shows a more horizontal arrangement, whereas in Fig. 3C a more vertical alignment is seen. The very same mouse liver lobule was also investigated histologically. The stained mouse liver lobule was prepared according to standard protocols and cut into very thin microscopic slices (3 µm). Figure 3D shows a light microscope image of the tissue sample directly after cutting without further processing, i.e. no staining was applied by the histologist. The histological image confirmed the staining of the hepatocytes and other cells such as Kupffer cells and SECs, which appear here in dark purple. The BC network is shown in white.

### 3D visualization and analysis

**Figure 4:**
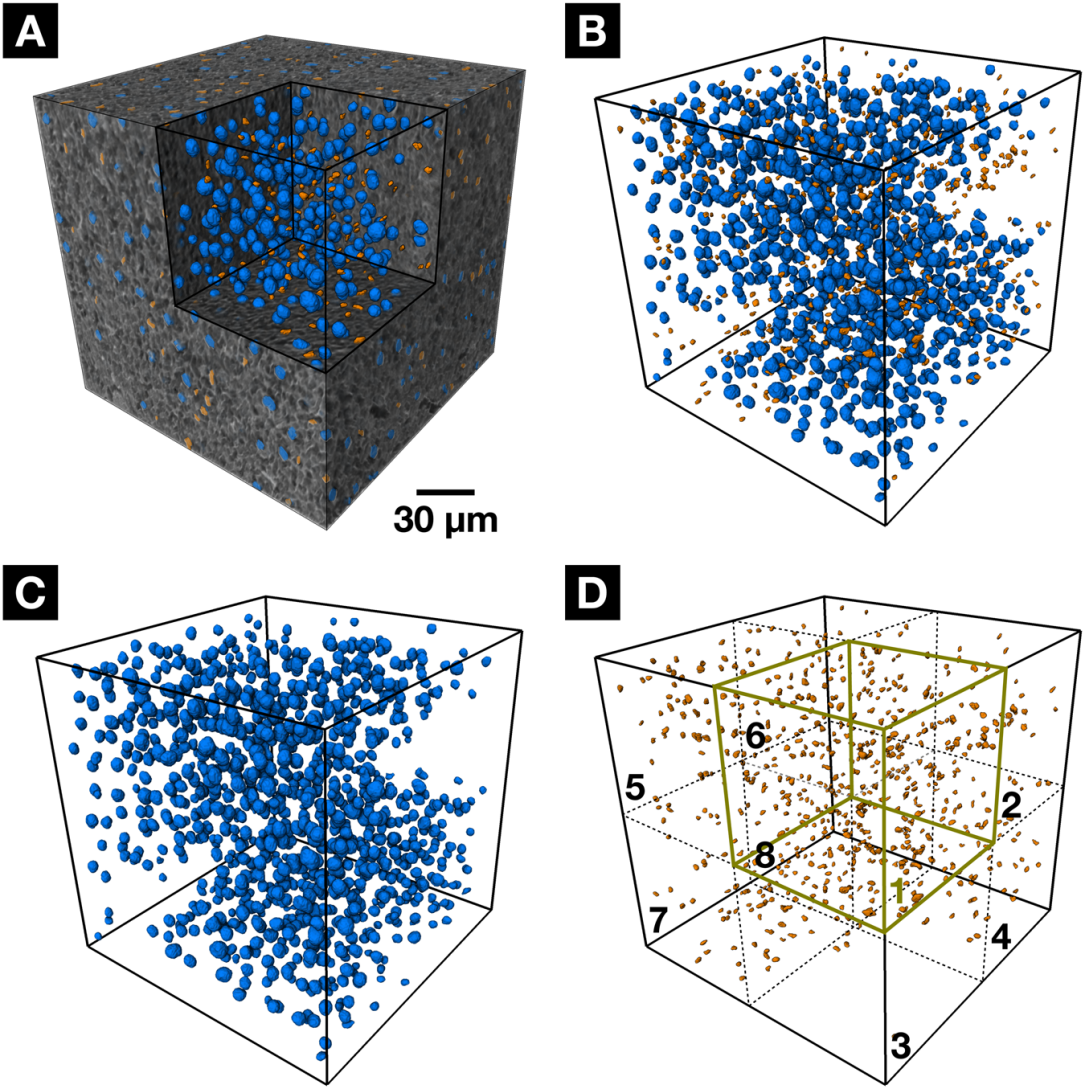
3D visualization and analysis of the different cell nuclei present within the mouse liver VOI. (**A**) 3D VOI showing the segmentation (inner volume and segmented cross-section of the cube surfaces) of all the cell nuclei from the original gray-value nanoCT data with hepatocytes labeled in blue and the other cells (containing Kupffer cells and SECs) highlighted in orange. (**B**) Entire segmentation (only inner volume without segmented cross-section of the cube surfaces) of all hepatocytes (shown in blue) and other cells such as Kupffer cells and SECs (shown in orange) is shown. (**C**) 3D distribution of the hepatocytes within VOI. (**D**) 3D distribution of all other cell nuclei within VOI. For 3D analysis the VOI has been compartmentalized in eight sub-cubes, whereby each sub-cube is visualized by dotted lines and numbered accordingly. Sub-cube one is highlighted in green for clarity. A movie is provided in the supplementary information (Movie S1).

The enormous potential of CT technology, the 3D visualization and analysis of the 3D data, is demonstrated in Fig. 4. A volume of interest (VOI) has been chosen for cell nuclei visualization and distribution analysis within this VOI. The VOI is the same as shown in Fig. 3. Next to the hepatocytes and other cell types containing Kupffer cells and SECs, the VOI contains a portal vein, which for clarity reasons has been omitted. Due to differences in attenuation values, size and shape, the nuclei could be separated efficiently from the surrounding tissue by using standard segmentation methods. Quantitative morphological analyses of the segmented data allowed for a classification into two different cell type categories (Fig. 4B–D). A comparison with regards to total cell numbers to a 3D data set obtained from a stitched confocal microscopic data set^20^ and a 2D microscopic data set^21^ is provided. The good agreement with the reconstructed 3D data from Morales-Navarrete *et al*.^20^ highlights once more the significance of accurate 3D reconstructions. For further 3D analysis of the cell nuclei distribution, the VOI was compartmentalized in further eight sub-cubes of approximately (100 × 100 × 100) µm³ in size. Due to the presence of the portal vein in the sub-cubes 2, 6, 7 and 8, they contain less cell nuclei compared to the other sub-cubes. Looking at the individual sub-cubes revealed a strong deviation of the ratio of hepatocyte cell nuclei to other cell nuclei for sub-cube 8. A summary of the results is displayed in Table 1.

**Table 1:** 3D VOI (see Fig. 4 and Movie S1) analyses of individual sub-cubes 1 to 8 including comparison to 3D and 2D microscopic data reported in scientific literature^20,21^.

Sub-cube	No. of hepatocytes	No. of other cells^a^	No. of all cells	% Hepatocytes
1	178	82	260	68.5
2	112	90	202	55.4
3	172	53	225	76.40
4	165	81	246	67.1
5	170	100	270	63.0
6	113	91	204	55.4
7	88	82	170	51.8
8	41	111	152	36.9
total^b^:	1039	690	1729	60.1
total^c,^^20^	1326 933.1^d^	805 566.5^d^	2131 1499.6^d^	62.2 62.2^d^
total^e,^^21^	66.8 951.6^f^	83 1182.3^f^	149.8 2133.9^f^	44.6 44.6^f^

^a^Other cells such as Kupffer cells and SECs. ^b^A VOI of (200 × 200 × 200) µm³ was studied. ^c^Hernán Morales-Navarrete *et al*. investigated a high-resolution VOI holding the dimensions of (300 × 300 × 300) µm³ ^20^. ^d^Cell numbers were corrected by 3.375 to reach comparable size of VOI (200 × 200 × 200) µm³. ^e^Baratta *et al*. identified relative numbers of cells by studying sets of 12 µm thick sections with an area of 46,800 µm² [(260 × 180) µm²] (40x magnification lens used), which represents 561,600 µm³ ^21^. ^f^Cell numbers were corrected to reach comparable size of VOI (200 × 200 × 200) µm³.

### Histological compatibility

**Figure 5:**
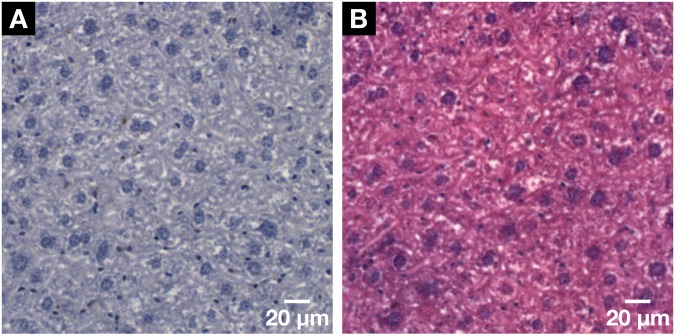
Demonstration of the histological compatibility of the for X-ray microCT and nanoCT developed hematein-based staining method with conventional 2D histology. (**A**) Representative histological microscopic slide with a thickness of 3 µm obtained from the same mouse liver lobule sample after the applied hematein-based staining and embedding in a paraffin block. Clear visualization of the larger hepatocyte cell nuclei and the smaller cell nuclei such as Kupffer cells and SECs in dark purple and the BC network displayed in white. (**B**) The compatibility with the standard counter stain of eosin Y was shown on a subsequent microscopic slide seen in (**A**). The cell nuclei are shown in purple next to the cytoplasm in pink resulting in a typical H&E stained microscopic slide of a soft-tissue sample.

To allow for an even better comparison with the histological results and showcase the compatibility of the developed hematein-based staining protocol with standard histological methods (Fig. 5A), the cytoplasm-specific staining with the counter stain eosin Y was applied to the histological microscopic slide (Fig. 5B). As expected the cell nuclei appear still purple in color and the hematein staining was not disturbed by the additional eosin Y stain following standard histological H&E staining procedures.

## Discussion

The low intrinsic attenuation properties of soft tissue for typically used X-ray energies of laboratory-based microCT systems, which consists of mainly carbon, hydrogen, oxygen and nitrogen^22^, provide an explanation for no contrast enhancement of unstained mouse liver tissue. As for the stained mouse liver lobules, the low concentration and attenuation properties of the hematein metal complexes used for staining with the standard histological protocols were the limiting factors. In the case of Mayer’s hematoxylin^23^, high atomic number elements are missing completely as the metal ion aluminum (Z = 13) has a low atomic number Z. When looking at Weigert’s iron hematoxylin^24^, sensitivity levels were not met for X-ray CT imaging with regards to the high atomic number element iron (Z = 26)^22^, of which one iron atom holds at least one hematein ligand (Supplementary Fig. S1 and staining protocols used). All the requirements for a complete and homogeneous staining enabling CT contrast enhancement of a whole mouse organ such as a mouse liver lobule (Fig. 2) were met for soft-tissue samples prepared with the hematein-based staining protocol. Throughout the soft-tissue sample, the preservation of the morphology is given to identify important anatomical regions and structures, which can be assigned by a pathologist. Moreover, for microCT imaging prepared soft-tissue samples are suitable for further histological investigations, whereby the histological microscopic slides are derived from the very same stained soft-tissue samples (Fig. 3D). Our staining method does not impede further histological treatment by the pathologists and enables the direct evaluation of the tissue with an optical microscope and the counter staining with the eosin stain. The concentration of the staining solution is much higher compared to the staining solution used in histology. Nevertheless, the contrast enhancement of the histological microscopic slides is very well suited for histological studies (Figs. 3D and 5).

The staining properties of the hematein stain remain intact, which is seen by the visualization of the cell nuclei in the nanoCT data as bright attenuation area (Fig. 3A–C) and confirmed by histology (Figs. 3D and 5). Here, the cell nuclei appear purple, which reflects the localization of the hematein stain within the nucleus of the cell. Counter staining was demonstrated by applying the cell cytoplasm-specific eosin stain, which resulted in a H&E stained histological microscopic slide (Fig. 5B) displaying the expected form of appearance. Full compatibility is given, and quality of the staining result remains very high. Next to the cell nuclei, little but some background staining derived from the X-ray suitable hematein staining protocol (Fig. 3D). This can be explained with the nature of the hematein stain being not as specific such as other DNA-like stains such as DAPI, which work on intercalation^25^. The background staining results from the interaction of the lead(II)-hematein complex with negatively charged (sub)-cellular components even in an very acidic environment. Mostly, this refers to DNA, but not exclusively.

The hematein-based staining protocol allows for high-resolution CT visualization specifically for cell nuclei within soft tissue down to the sub-micron range, which has not been possible so far applying other common staining methods used in microCT technology^11–16^. Nondestructive generation of virtual histological slices that are comparable in contrast and resolution to conventional histological data is rendered possible in combination with the recently developed nanoCT devices^6–10,26–28^. In the future, the elaborate and time-consuming preparation of individual slices required for standard histology can be bypassed for those pathological samples (e.g. biospies), where a 3D investigation of an entire volume as provided by the nanoCT is of great benefit and sectioning of the specimen will result in e.g. an information loss. Out of the obtained 3D data set, the pathologist will be enabled to directly identify anatomical structures and regions of interest. Furthermore, this method provides real 3D information of a soft-tissue bulk sample that supports the pathologist with additional information regarding anatomical structures, which may facilitate improved diagnosis. In particular, it allows for a quantitative 3D analyses such as 3D distribution of cell nuclei within a VOI. Once more specific segmentation and classification algorithms have been developed, traditional and spatial statistical analysis of tissue are rendered possible in an efficient, time-saving and nondestructive way. This may lead to insights into tissue organization from the geometrical model, which feature important implications for the development of models of fluid exchange between blood and hepatocytes^20^. Morphometric parameters of the liver tissue are often limited, and here, 3D microCT and nanoCT data can help to understand these better such as the heterogeneity present within liver tissue. Furthermore, a screening of larger samples for abnormalities in local cell nuclei distribution should identify regions of inflammation or help to understand diseases (e.g. cancer) where abnormalities in local cell nuclei distribution are crucial.

Our staining procedure is simple to apply and suitable for a whole-organ CT staining, which enables 3D visualization and analysis of soft-tissue samples (Fig. 4). The staining agents or other reagents involved in the staining protocol did not affect the soft-tissue sample size. Even though a comparison of our results with other microscopic data (Table 1) was in good agreement, the absolute numbers of cell density have to be taken with care as we do not know any aspects regarding the soft-tissue shrinkage with respect to the microscopic data. Therefore, an absolute comparison is not possible, and Table 1 has to be seen as a first attempt to highlight the benefit of a 3D analysis over a 2D analysis.

The required staining agents are easily accessible. Thus, for pathological soft-tissue samples, overview scans will provide valuable insights into altered anatomical regions and structures, which allow for the determination of regions of interest. 3D investigations by microCT or nanoCT can be further evaluated in 2D with histology. Further counter staining remains possible. As in conventional 2D histology hematoxylin and eosin (H&E) are always applied in combination, the development of a combined X-ray suitable H&E staining protocol will allow much more sophisticated soft-tissue sample analyses. Together with spectral CT imaging techniques such as dual energy CT^29^ or the use of hyperspectral detectors^30^, double-labelling of soft-tissue samples using a combined H&E staining protocol will be lifted to the next level. However, the double-labelling of soft-tissue samples adds a lot more complexity to the staining process compared to a single-labelling procedure. From a chemical perspective, both developed methods involve acidification of the soft-tissue sample during fixation or prior to staining. This is a good prerequisite for the development of a combined H&E staining protocol, as the soft tissue needs the same pH conditions. However, pH of the soft tissue is just one aspect, which needs to be looked at when developing a combined H&E staining protocol. Further parameters such as the order of the staining steps, influence of buffer solutions or incubation times need to be systematically analyzed.

Next to the chemical aspects, we have to consider the technical requirements for spectral X-ray imaging. To separate the contributions of an H&E-stained sample by this kind of measurements, the attenuation properties of the individual stains have to be considered. For eosin Y, the attenuation is mainly given by the attenuation coefficient of bromine, which has its K-edge at 13.48 keV. For the hematein staining, the attenuation is dominated by the attenuation coefficient of lead, which has its K-edge at 88.01 keV. For the typically used energies of laboratory-based microCT and nanoCT devices, the most straight-forward approach to guarantee a significant difference in the absorption for dual energy measurements would be to select the corresponding spectra in such a way that only the low-energy spectrum includes substantial contributions from energies below the K-edge of bromine.

Overall, this work on 3D X-ray histology highlights microCT and nanoCT technology as a modern tool for future histological and histopathological applications, which can be achieved with laboratory devices.

## Conclusions

The histological staining method based on hematoxylin exists in many different variations, of which two have been applied using the standard protocol. The missing contrast enhancement led to the development of an X-ray tailored staining protocol. By using a well-directed chemical approach acknowledging the biological, technological and histological requirements, the hematein-based X-ray staining method offers a first approach for the specific targeting of the cell nuclei in 3D. Several benefits such as the nondestructive access to a series of CT slices in arbitrary planes and the 3D visualization and analysis of a histological bulk soft-tissue sample allow for fundamental insights into geometric and morphometric parameters of the sample. Furthermore, full compatibility of the method with 2D conventional histology, makes microCT and nanoCT a well-suited tool for modern histopathological investigations. A first approach in 3D analysis was demonstrated highlighting the enormous potential of microCT and nanoCT technology in combination with the hematein-based staining method, which may assist in the understanding of widespread diseases such as osteoarthritis and cancer. To further improve soft-tissue analyses using X-ray imaging approaches, the development of a combined H&E staining protocol in conjunction with a concept for spectral X-ray imaging sets a great future perspective for the field of X-ray imaging and its many applications such as 3D histology or developmental and structural biology.

## Materials and Methods

### Animals Used

The animals were housed at the Klinikum rechts der Isar, Technical University of Munich in conformity with institutional guideline. The internal animal protection committee of the Center for Preclinical Research (ZPF) of Klinikum rechts der Isar, Munich, Germany approved the post mortem organ removal (internal reference number 4-005-09). All procedures were in accordance with the Guide for the Care and Use of Laboratory Animals published by the U.S. National Institutes of Health (NIH publication No. 85-23, revised 1996). All laboratories are inspected for agreement with the Organization for Economic Co-operation and Development (OECD) principles of good laboratory practice. We prepared a whole mouse liver lobule using the final version of the hematein-staining procedure. Investigations of the soft-tissue sample were carried out to review structural preservation and to evaluate stain quality, identify morphological structures, compare with conventional histological methods and assess for further histological staining. The development of the staining protocol and the optimization of parameters was performed with the remaining organs.

### Sample Screening

We purchased all reagents from Sigma-Aldrich unless otherwise indicated. Fixation and preservation of the whole mouse organs was performed under the conditions described below. Stain development and optimization was carried out with cuboidal soft-tissue samples from mouse liver (2-3 mm edge length), which were cut with a scalpel (Aesculap). All samples were kept under controlled temperature conditions by placing samples in a refrigerator (4 °C) or in ambient conditions of the laboratory. Incubations were done in sample holders with a flat bottom, which were replaced after each step but not after rinse or dehydration steps. Several parameters were investigated regarding stain development and optimization: (i) type of fixative, (ii) concentration of fixative, (iii) concentration of staining agents, (iv) incubation times, (v) pH of fixative, or (vi) pH of staining agents to name a few. MicroCT scanning of the stained soft-tissue samples were realized on the phoenix v|tome|x s 240 CT scanner with typical settings of 50 kV peak voltage, 6.0 W and with 1001 projections distributed over 360°. An exposure time of 1 s per projection with an effective pixel size of approximately 30 µm was used for low resolution CT data acquisition. Reconstruction of the microCT data was performed with the integrated phoenix datos x CT software. Analyses focused on parameters such as (i) completeness of staining, (ii) appearance of diffusion rings, (iii) contrast enhancement, (iv) appearance of CT artifacts as streaks and (v) homogeneity of the staining.

### Preparation of staining working solutions

#### Staining Solution (A)

Lead(II) acetate trihydrate (3,7896 g, 9.99 mmol; Merck Millipore) was dissolved in 15 ml dist. water to yield an aqueous lead(II) acetate solution (c = 0.666 M). The solution was prepared fresh for each experiment and was used maximum up to 4 weeks past preparation.

#### Staining Solution (B)

Hematoxylin (25 g, 82.7 mmol) was dissolved in 250 ml absolute ethanol (10% (w/v), c = 0.333 M). During dissolving the reaction flask was kept in a water bath and the temperature was set to 56 °C. The reaction mixture was kept stirring for one month under ambient laboratory conditions to allow the oxidation of hematoxylin to hematein. The ripened hematein solution was kept in the dark for up to one year past preparation in an air-tight container (Schott Bottle).

### Hematein Staining Protocol

The mouse organ was surgically removed and immediately placed in a 50-ml Falcon Centrifuge Tube (neoLab), which was filled with a fixative solution containing 9.5 ml of 4% (v/v) formaldehyde solution (FA, derived from a 37% acid free FA solution stabilized with 10% methanol from Carl Roth; further dilution with DPBS without calcium and magnesium) and 0.5 ml glacial acetic acid (AA, Alfa Aesar). The sample was refrigerated for 24–72 h and then washed with phosphate saline buffer solution for 1 h (DPBS without calcium and magnesium). The mouse organ was placed in the staining solution (A). The soft-tissue sample was stained with 3 ml of staining solution (A) for 72 h (the soft-tissue sample was moving freely within the sample container). During the incubation time the soft-tissue sample was kept in the dark and on a horizontal shaking plate allowing for a smooth rocking of 60 rpm. After the first staining step, the soft-tissue sample was carefully removed and placed for a short time period in an Eppendorf tube above an ethanol vapor phase to keep the soft-tissue sample moist (Eppendorf tube contained a few drops of 70% (v/v) ethanol at the bottom of the tube). In the meantime, 3 ml of working solution (B) were added to the same sample container holding working solution (A), followed by thorough stirring of the reaction mixture. Immediate color change to deep purple upon addition of staining solution (B) was observed. The soft-tissue sample was placed back into the sample container and incubated for further 72 h. During the incubation time the soft-tissue sample was kept in the dark and on a horizontal shaking plate allowing for a smooth rocking of 60 rpm. After staining the soft-tissue sample was carefully removed from the sample container and access of staining agent was softly patted with a cellulose tissue paper. The soft-tissue sample was washed with 6 ml tab water (washing solution was changed every hour for the first 5 hours) and kept overnight in the washing solution. During the incubation time the soft-tissue sample was kept in the dark and on a horizontal shaking plate allowing for a smooth rocking of 60 rpm. The soft-tissue sample was stored in an Eppendorf tube above an ethanol vapor phase (the Eppendorf tube contained a few drops of 70% (v/v) ethanol at the bottom of the tube).

### X-ray microCT Imaging

The stained mouse liver lobule was transferred to a sample holder, which allows the anchorage of the mouse organ above 70% (v/v) ethanol vapor. The X-ray microCT measurements were performed with the ZEISS Xradia Versa 500. All shown images were acquired at 50 kV acceleration voltage, 3.5 W and with 1601 projections evenly distributed over 360°. The low-resolution CT data were acquired with the 0.39x objective and an exposure time of 2 s per projection with an effective pixel size of 13.5 µm.

### Sample Preparation for nanoCT

The stained mouse liver lobule was dehydrated and critically point dried (CPD) prior to nanoCT imaging. Before the first step, a fifth of the stained mouse liver lobule was cut off and further sectioned for nanoCT imaging analysis. The tissue pieces had a size of approximately 0.5 mm edge length. The dehydration incubations were performed for 1 h each. Before the first dehydration step, the small pieces were transferred to a new petri dish, where they remained for all subsequent steps. The used concentrations (all v/v) for the dehydration series were in %: 50, 60, 70, 80, 90, 96 and 100 ethanol balanced with dist. water. The dehydrated mouse tissue pieces were then CPD using a Bal-TEC CPD 030 with CO_2_ as drying agent. The CPD mouse tissue pieces were stored in a petri dish kept in a desiccator prior to further use.

### X-ray nanoCT Imaging and data analysis

The X-ray nanoCT measurements were performed with an in-house developed nanoCT system that comprises of nanofocus X-ray source (prototype NanoTube, Excillum, Sweden)^23^, a rotation stage with a sample holder and a single-photon counting detector (PILATUS 300K-W 20 Hz, Dectris, Switzerland)^24,25^. The system does not include any X-ray optics and can efficiently generate 3D data with resolutions down to 100 nm^10^. Since the field of view for a single CT scan is limited to approximately 1,400 voxels in the horizontal direction and 190 voxels in the vertical direction, the liver piece was imaged by performing three separate CT measurements at different vertical positions and subsequently combining them to a single volume. Each CT measurement was acquired at an acceleration voltage of 60 kV with 1599 projections evenly distributed over 360° and a voxel size of approximately 580 nm. The exposure time per image was 1 s and the total acquisition time per dataset was ∼1.5 h. The volume data were reconstructed with a state-of-the-art filtered backprojection algorithm and a representative VOI was selected for further analysis.

The segmentation and pre-classification of the cell nuclei was performed with the interactive learning and segmentation toolkit ilastik 1.2.2^31^. A small number of representative examples for the three chosen object classes, i.e., the nuclei of the hepatocytes, the nuclei of the smaller cells and the background, were manually selected in a few slices of the volumetric dataset and the classifiers were trained using different feature types of the objects, namely, the intensity, edges and textures of the nuclei. Subsequently, all pixels of the dataset were automatically classified into one of the three classes. To further improve the classification results, Avizo Fire 8.1 (FEI Visualization Sciences Group, Burlington, MA, USA) was used to perform a three-dimensional morphological classification with respect to size and shape of the segmented nuclei. Finally, the volume renderings in Figs. 3, 4 and Movie 1 were generated using Avizo Fire 8.1.

### Histological Analysis

After CT analysis, the mouse liver lobule was dehydrated and embedded in paraffin according to standard procedures. Briefly, the soft-tissue sample was gradually dehydrated by an ascending gradient of ethanol (50%, 60%, 70% and 96%) twice for 1 h, cleared in xylol twice for 1 h and subsequently, embedded in paraffin wax. Sections of 3 µm and 7 µm thickness were cut using a microtome (Leica). Sections were rehydrated and either directly embedded (Eukitt, Merck) or counterstained for 5 min with eosin Y solution (Morphisto) according to the manufacturer’s protocol. Histological analysis was performed using an Axio Imager 2 microscope and AxioVision Software (Zeiss).

## Electronic supplementary material

Supplementray Information

Movie S1
