# Author Correction: Nucleus-specific X-ray stain for 3D virtual histology

**DOI:** 10.1038/s41598-019-42416-2

**Published:** 2019-04-18

**Authors:** Mark Müller, Melanie A. Kimm, Simone Ferstl, Sebastian Allner, Klaus Achterhold, Julia Herzen, Franz Pfeiffer, Madleen Busse

**Affiliations:** 1 Department of Physics and Munich School of BioEngineering, Technical University of Munich, 85748 Garching, Germany; 2 Department of Diagnostic and Interventional Radiology, Klinikum rechts der Isar, Technical University of Munich, 81675 Munich, Germany

Correction to: *Scientific Reports* 10.1038/s41598-018-36067-y, published online 14 December 2018

This Article contains errors.

In Figure 1A, the final step of the Hematein staining protocol is incorrectly labelled. The correct Figure 1 appears below.

**Figure 1:**
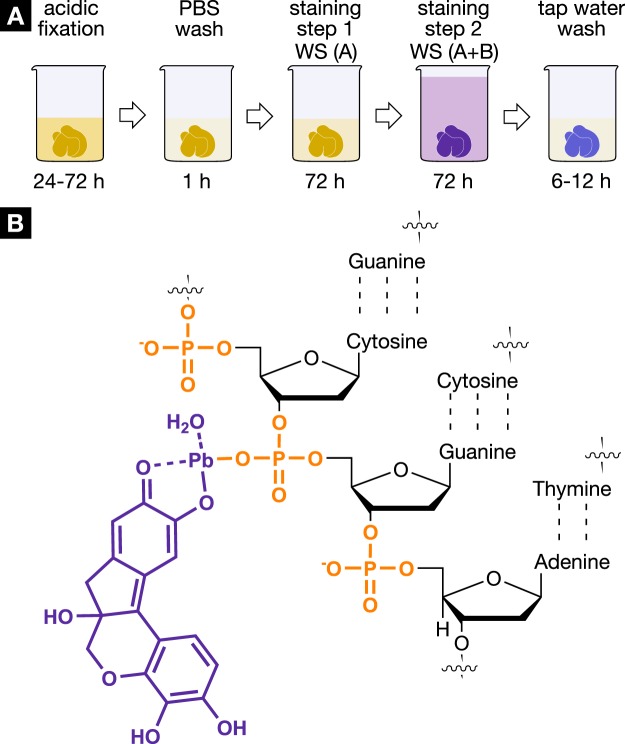
Staining protocol and interaction of the hematein-based X-ray stain with soft tissue. (**A**) The developed hematein-based staining procedure shows the individual steps involved including incubation and staining times. Staining step 1 was conducted using lead(II) acetate trihydrate as the heavy metal source. The lead(II) acetate trihydrate was dissolved in distilled water (c = 666 mM) and is referred to as working solution (A) (WS (A)). The staining step 2 involved a hematein solution in absolute ethanol (WS (B), 10% (w/v); c = 333 mM), which was derived from hematoxylin and was added to WS (A). More details concerning the entire staining protocol are described in the Materials and Methods section. (**B**) The positively charged hematein lead(II) complex (purple), which is built in situ in the soft-tissue sample, is interacting with the negatively charged phosphate backbone of the DNA (orange) present in the nucleus of the cell. The selective interaction of the hematein lead(II) complex with the DNA is achieved by acidification of the soft tissue during fixation or before staining and allows for a higher accumulation of the hematein lead(II) complex within the cell nucleus.

Additionally, this Article contains a typographical error in the Results section, under the subheading ‘MicroCT investigations of hematein stained mouse liver tissue’ where,

“The tab water wash after staining is crucial for the specific cell nuclei staining result.”

should read:

“The tap water wash after staining is crucial for the specific cell nuclei staining result.”

This Article also contains a typographical error in the Methods section, under the subheading ‘Hematein Staining Protocol’ where,

“The soft-tissue sample was washed with 6 ml tab water (washing solution was changed every hour for the first 5 hours) and kept overnight in the washing solution.”

should read:

“The soft-tissue sample was washed with 6 ml tap water (washing solution was changed every hour for the first 5 hours) and kept overnight in the washing solution.”

Lastly, the Article contains an error in Table 1 where the incorrect % Hepatocytes value is given for Sub-cube 8. The correct value should read 27.0.

