# Supplementary material for: Nucleus-specific X-ray stain for 3D virtual histology
Source: Sci Rep. 2018 Dec 14;8:17855. doi: 10.1038/s41598-018-36067-y (PMC6294809; doi:10.1038/s41598-018-36067-y)
Supplement: Supplementary file 1 — Supplementray Information [file 41598_2018_36067_MOESM1_ESM.pdf]

Supplementary Information

Title: Nucleus-specific X-ray stain for 3D virtual histology

Authors: Mark Müller[1,#,*], Melanie A. Kimm[2,#], Simone Ferstl[1], Sebastian Allner[1], Klaus Achterhold[1], Julia Herzen[1], Franz Pfeiffer[1,2] and Madleen Busse[1,*]

Affiliations:

[1]Department of Physics and Munich School of Bioengineering, Technical University of Munich, 85748 Garching, Germany.

[2]Department of Diagnostic and Interventional Radiology, Klinikum rechts der Isar, Technical University of Munich, 81675 Munich, Germany.

[#]M.M. and M.A.K. contributed equally to this work.

*Correspondence to: madleen.busse@tum.de and mark_mueller@ph.tum.de

Supplementary Materials:

Staining Protocol according to histological hematoxylin-based procedures:

(a) Hematoxylin according to Mayer

The mouse organ was removed post mortem and immediately placed in a 50-ml Falcon Centrifuge Tube (neoLab), which was filled with a fixative solution containing 15 ml of 4% (v/v) formaldehyde solution (FA, derived from a 37% acid free FA solution stabilized with ca. 10% methanol from Carl Roth; further dilution with DPBS without calcium and magnesium). The sample was refrigerated for 24-72 hours and then washed with phosphate saline buffer solution for 1hour (DPBS without calcium and magnesium). The mouse organ was placed in Mayer's staining solution (Morphisto). The soft-tissue sample was stained with 6 ml of Mayer's staining solution for 72 hours (the soft-tissue sample was moving freely within the sample container). During the incubation time the soft-tissue sample was kept in the dark and on a horizontal shaking plate allowing for a smooth rocking of 60 rpm. After staining the soft-tissue sample was carefully removed from the sample container and access of staining agent was softly patted with a cellulose tissue paper. The soft-tissue sample was washed with 6ml tab water (washing solution was changed every hour for the first 5 hours) and kept overnight in the washing solution. During the incubation time the soft-tissue sample was kept in the dark and on a horizontal shaking plate allowing for a smooth rocking of 60 rpm. The soft-tissue sample was stored in an Eppendorf tube above an ethanol vapor phase (the Eppendorf tube contained a few drops of 70 % (v/v) ethanol at the bottom of the tube).

(b) Hematoxylin according to Weigert

Organ preparation was performed as described in a). The mouse organ was placed in Weigert's staining solution (Working solution A and B were mixed in the ratio of 1/1 prior to use; Morphisto). The soft-tissue sample was stained with 6ml of Weigert's staining solution for 72 hours (the soft-tissue sample was moving freely within the sample container). During the incubation time the soft-tissue sample was kept in the dark and on a horizontal shaking plate allowing for a smooth rocking of 60rpm. After staining the soft-tissue sample was treated as described in a).

(c) Hematoxylin according to Weigert applying the stepwise staining procedure

Organ preparation was performed as described in a). The mouse organ was placed in Weigert's staining solution (B) (Morphisto). The soft-tissue sample was stained with 3ml of Weigert's staining solution (B) for 72 hours (the soft-tissue sample was moving freely within the sample container). During the incubation time the soft-tissue sample was kept in the dark and on a horizontal shaking plate allowing for a smooth rocking of 60rpm. After the first staining step, the soft-tissue sample was carefully removed and placed for a brief time period in an Eppendorf tube above an ethanol vapor phase (Eppendorf tube contained a few drops of 70% (v/v) ethanol at the bottom of the tube). Addition of 3ml of Weigert's staining solution (A) (Morphisto) to the sample container was performed, followed by thorough stirring of the reaction mixture. Immediate color change to purple upon addition of staining solution (A) was observed. The soft-tissue sample was placed back into the sample container and incubated for further 72 hours. During the incubation time the soft-tissue sample was kept in the dark and on a horizontal shaking plate allowing

for a smooth rocking of 60rpm. After staining the soft-tissue sample was treated as described in a).

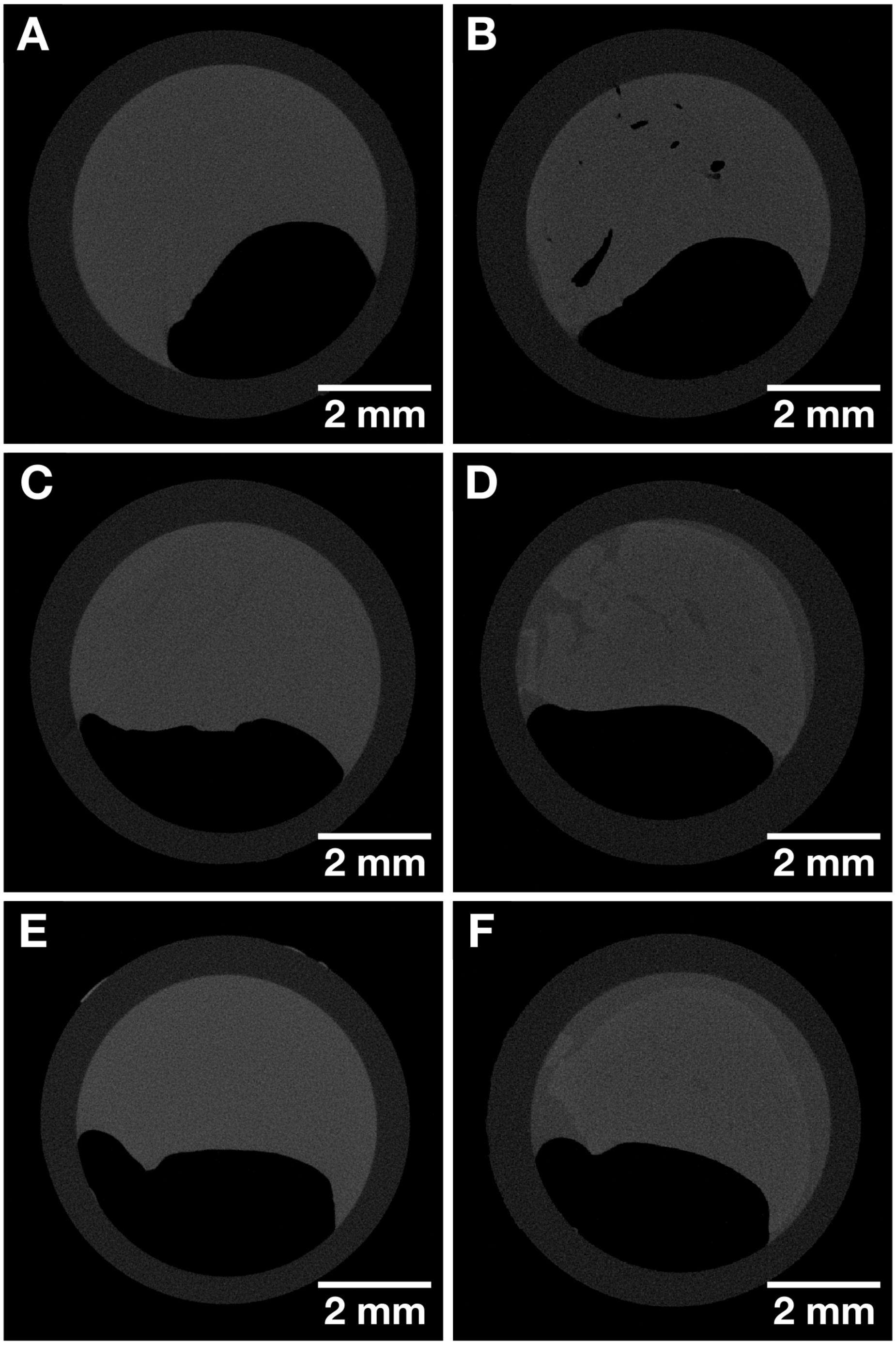

Figure S1. CT slices (xy plane) of the same whole mouse liver lobule before and after staining following standard histological staining protocols. The Xradia Versa 500 microCT was used to acquire all data sets under identical acquisition parameters. The voxel size in all data sets is around 14µm. (**A,B**) Overview images of an (**A**) unstained and (**B**) the very same stained mouse liver lobule with hematoxylin according to Mayer (*15*). (**C,D**) Overview images of an (**C**) unstained and (**D**) the very same stained mouse liver lobule with hematoxylin according to Weigert (*16*). (**E,F**) Overview images of an (**E**) unstained and (**F**) the very same stained mouse liver lobule with hematoxylin according to Weigert following the stepwise approach of the developed hematein-based staining procedure.

Movie S1: Animation showing 3D distribution of cell nuclei within the VOI. For 3D analysis the VOI has been compartmentalized in eight sub-cubes, whereby each sub-cube is visualized by dotted lines and numbered accordingly. Sub-cube one is highlighted in green for clarity.

Table S1: Parameters and conditions tested for the development and optimization of the X-ray suitable hematein staining protocol.

Parameters tested	Conditions tested
**fixative**	1% FA[1], 4% FA[1]; mixture: 1 % FA[1]; 2.5 % GA[2]
**pH**	with / without glacial acetic acid
**concentration of $Pb(OAc)_2$ solution**	333 mM, 666 mM
**incubation time**	6 h – 1 week (for individual steps each)
**staining procedure**	**1 step protocol:**<br>working solutions A[3] and B[4] were freshly mixed prior to use<br><br>**2 step protocol:**<br>i) 1st working solution A[3] and 2nd addition of working solution B[4] into the same sample container<br>ii) 1st working solution B[4] and 2nd addition of working solution A[3] into the same sample container<br>iii) 1st working solution A[3] and 2nd working solution B[4] (new sample container)<br>iv) 1st working solution B[4] and 2nd working solution A[3] (new sample container)

[1]FA = formaldehyde; [2]GA = glutaraldehyde; [3]working solution A: hematoxylin solution in absolute ethanol; [4]working solution B: lead(II) acetate trihydrate solution in dist. Water.
